# Identification and Analysis of Weather-Sensitive Roads Based on Smartphone Sensor Data: A Case Study in Jakarta

**DOI:** 10.3390/s21072405

**Published:** 2021-03-31

**Authors:** Chao-Lung Yang, Hendri Sutrisno, Arnold Samuel Chan, Hendrik Tampubolon, Budhi Sholeh Wibowo

**Affiliations:** 1 Department of Industrial Management, National Taiwan University of Science and Technology, Taipei 106, Taiwan; d10701802@mail.ntust.edu.tw (H.S.); m10601803@mail.ntust.edu.tw (A.S.C.); 2 Department of Computer Science and Information Engineering, National Taiwan University of Science and Technology, Taipei 106, Taiwan; d10715806@mail.ntust.edu.tw; 3 Mechanical and Industrial Engineering Department, Universitas Gadjah Mada, Yogyakarta 55281, Indonesia; budhi.sholehwibowo@ugm.ac.id

**Keywords:** weather-sensitive road, smartphone sensor data, traffic congestion, spatial-temporal, clustering, classification, intelligent transportation system

## Abstract

Weather change such as raining is a crucial factor to cause traffic congestion, especially in metropolises with the limited sewer system infrastructures. Identifying the roads which are sensitive to weather changes, defined as weather-sensitive roads (WSR), can facilitate the infrastructure development. In the literature, little research focused on studying weather factors of developing countries that might have deficient infrastructures. In this research, to fill the gap, the real-world data associating with Jakarta, Indonesia, was studied to identify WSR based on smartphone sensor data, real-time weather information, and road characteristics datasets. A spatial-temporal congestion speed matrix (STC) was proposed to illustrate traffic speed changes over time. Under the proposed STC, a sequential clustering and classification framework was applied to identify the WSR in terms of traffic speed. In this work, the causes of WSR were evaluated based on the variables’ importance of the classification method. The experimental results show that the proposed method can cluster the roads according to the pattern changes in the traffic speed caused by weather change. Based on the results, we found that the distances to shopping malls, mosques, schools, and the roads’ altitude, length, width, and the number of lanes are highly correlated to WSR in Jakarta.

## 1. Introduction

Weather is an essential causing factor of traffic congestion, especially in metropolises of developing countries. Due to the limited infrastructure, such as flawed mass transport, deficient sewer systems, and relatively narrow roads, in developing countries, such as Vietnam or Indonesia [1,2,3], traffic is vulnerable when it rains. Meanwhile, the astronomical economic loss caused by the congestion highlights the urgency of the solutions. For example, traffic congestion leads to US$5 billion lost in Jakarta [4], US$11.4 billion in Dhaka [5], and US$18 billion in Metro Manila [6] annually. The tremendous economic loss underlines the importance of solving traffic congestion in the metropolises of developing countries.

Without a doubt, upgrading the infrastructure to improve traffic congestion is costly and time-consuming. In the literature, researchers have proposed alternative and short-term solutions to remind drivers about traffic congestion, such as understanding the traffic patterns on different weather conditions [7,8,9]. In intelligent transport system (ITS) research, the traffic prediction models try to predict future traffic congestion in terms of location and time. For example, in Taipei City, Taiwan, many traffic bulletin boards were installed on major roads to announce real-time information regarding the roads’ traffic situation and prediction under different weather conditions. Based on the real-time announcement, drivers can choose different routes when necessary to avoid traffic congestion [10]. Other ITS applications also include adaptive street lighting control [11] to predict the traffic flow using camera devices installed on Strinella Street in L’Aquila city, Italy. In this research [11], the traffic prediction model was used to provide information about traffic congestion and provide a more efficient energy consumption of traffic lighting devices. In the ITS research domain, researchers assessed the impact of weather on vehicles’ speed drop in major metropolises of developed countries which have relatively well-established infrastructures, such as Paris [12], Chicago [13], London [14], Beijing [15], Shenzhen [16], and Seoul [17]. However, in the literature, few studies have been reported for municipalities with poor or limited infrastructures, especially in developing countries.

This paper aims to fill the research gap in the traffic study by investigating how weather change affects Jakarta’s traffic condition, Indonesia’s largest metropolis but with the limited infrastructure of sawing and mass transportation system. In 2019, Jakarta’s population reached 11 million, with a population density of 16,882 people/km^2^ [18]. According to the Traffic Index ranking by TomTom^®^, Jakarta was ranked 10th in the world’s worst traffic in 2019 [19]. With the unbalanced annual growth of 8% and 0.01% of private cars and road lengths, respectively, Jakarta city expects to face heavier traffic in the future. On account of the severe traffic condition, the Jakarta Smart City Department considers roads with an average speed of 10 km/h is a traffic-free road, which is equal to the common jogging speed for most adults [20].

**Figure 1:**
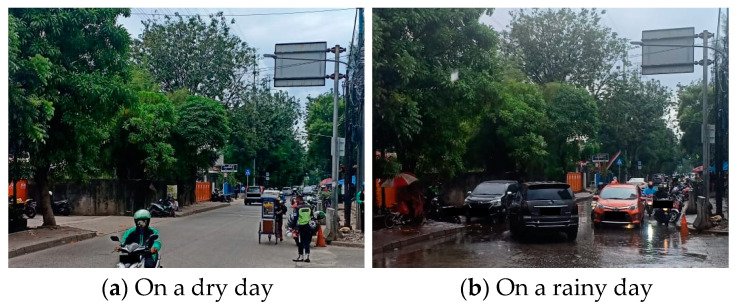
Pegangsaan Dua Road’s traffic condition in Jakarta, Indonesia, on dry (**a**) and rainy days (**b**). To avoid the bias, both photos were taken at the similar time. The photo (**a**) was taken on Thursday, 16 July 2020, at 2:30 p.m. while photo (**b**) was taken on Thursday, 13 August 2020, at 2:33 p.m. on exactly the same spot. No particular event was hosted nearby. The main difference between (**a**) and (**b**) is the weather condition.

Figure 1 illustrates how weather change causes traffic congestion at Jalan Pegangsaan Dua, Jakarta, Indonesia. Figure 1a,b show the traffic condition on dry and rainy days, respectively. This road is a representative example of many roads in Jakarta with no or inadequate sewer systems. As shown in Figure 1a, the road has mild traffic congestion on a dry day. However, many puddles are generated when it rains, as shown in Figure 1b. These puddles will eventually develop as potholes and create slippery roads. As a result, drivers tend to slow down to avoid a potential traffic accident, which ultimately creates traffic congestion on the road.

In this work, the real-world traffic speed data collected from citizens’ smartphones in Jakarta and daily weather data, especially for raining information, were utilized for traffic speed study. This paper also proposes the spatial-temporal congestion speed change matrix (STC) to identify the weather-sensitive roads (WSR) in Jakarta, whose traffic congestion dramatically suffers from weather changes, such as rain. The data analysis framework was presented under the proposed STC based on sequential clustering and classification methods [21] to identify and analyze the WSR. First, the K-means algorithm [22] was applied to cluster roads with similar STC patterns. Then, the random forests (RF) [23] were used to train the classifier for identifying the STC level, which measures the level of the traffic speed drop over time due to the weather change. The best number of clusters k was selected based on Pareto front [24], and the root causes of WSR were determined by using the RF method.

The experimental results show that using the data analysis framework under the proposed STC can identify the WSR in Jakarta. The results also present the significant driving factors regarding WSR: the spatial information of the road (the distances to public areas, such as shopping malls, mosques, or schools), road altitude, length, width, and the number of lanes. By utilizing the proposed framework, this work demonstrates the capability of tracing or investigating traffic conditions caused by weather change.

The rest of this paper is structured as follows. Section 2 discusses the related literature. Section 3 delivers the methodology. Section 4 shows the experimental results, and Section 5 concludes this study.

## 2. Literature Review

### 2.1. Weather Impact on Traffic Congestion

When evaluating the effect of weather change on vehicles’ speed drop, the popular method used in the literature is regression and trend analysis [7,15,25,26]. Camacho et al. [7] modeled the traffic speed of 15 major freeways in northern Spain using regression analysis based on several indicators: number of trucks, visual visibility, wind speed, precipitation intensity, and snow thickness. The results showed that the tested indicators were significantly affecting the traffic speed drop, mainly the precipitation intensity and the wind speed. Zhang et al. [15] applied the regression analysis to investigate the impact of rainfall on the traffic flow intensity in major expressways in Beijing, China. They found that different rainfall intensity affects traffic flow, as summarized in Table 1. The regression and analysis approaches were also used by Mitsakis et al. [25] and Stamos et al. [26].

Moreover, Billot et al. [12] proposed a multilevel assessment using microscopic, mesoscopic, and macroscopic approaches. The mesoscopic approach focused on understanding the drivers’ behavior under the adverse weather condition. Based on the insight at the microscopic level, they observed the platooning effect at the mesoscopic level. The global view to the weather effect on the traffic density and speed drop was carried out at a macroscopic level. Other researchers also applied the regression analysis method [8,14] and other statistical methods, such as the Gaussian mixture model [27], to study the weather impact on traffic flow or speed. Besides the statistical methods, researchers also applied a variety of machine learning methods for traffic studies, such as the *k*-means clustering method [28], neurowavelet models [29], long short-term memory network [30], Bayesian networks model [31], deep belief networks [32], and decision tree [9]. The work above demonstrates the promising performance of the machine learning method for the traffic problem.

Table 1 summarizes the relevant studies in the literature regarding the effect of rainy weather on vehicle speed reduction. As can be seen in Table 1, most of the literature studied traffic conditions in Western countries, such as the United States [13,33], France [12], Spain [7], Greece [25,26], Australia [31], and Sweden [34]. A few studies reported on the main metropolises in Asia, such as Hong Kong [8], Beijing [15], Shenzhen [16], and Seoul [17]. All of the mentioned works were conducted based on modern cities in developed countries. Very few studies pay attention to the weather impact on the developing country, whose traffic condition is more vulnerable due to the poor infrastructure.

Additionally, in terms of the road type, most of the works in Table 1 studied the high-speed vehicular traffic roads, such as freeways [7,12,33], highways [13,31], motorways [14], and expressways [15,16]. The speed of vehicles on high-speed vehicular traffic roads can be easily measured by loop detectors, road-side cameras, and on-board equipment. However, measuring vehicle speed in an urban area might need different technologies, such as wireless sensor networks. Although some researchers also focused on traffic studies on urban roads, all of those works considered the developed countries’ road condition, such as in South Korea [17], Sweden [33], the United States [35], Athens, Greece [25], and Thessaloniki, Greece [26]. In summary, each work mentioned above shows various weather impact with different precipitation and characteristics. For example, in Seoul, the study shows that weather change can reduce the traffic speed up to 50% [17]. Other results of the studies above are summarized in Table 1.

Moreover, considering urban roads’ condition in developing countries where various transportation modes—cars, trucks, motorcycles, pedestrians, and even wild animals—can be present on roads, measuring the roads’ traffic speeds is extremely difficult. In this research, the traffic data collected from citizens’ smartphones in Jakarta, Indonesia, was used to represent the traffic speed of a developing country’s urban roads. The weather data associated with Jakarta City was also collected for this research work. More details regarding the collected dataset will be presented in the following sections.

### 2.2. Machine Learning Method in Traffic Studies

In the literature, researchers have applied the *k*-means clustering algorithm [22] widely, especially in the traffic field. Liu [36] implemented the *k*-means clustering method to obtain the optimal system design of sensors in a complex coordination control in ITS. The ITS problem studied by [36] combines the issues of coordinating the traffic signal lamp, balancing the traffic flow, and reducing the travel time. Pattanaik et al. [37] used the *k*-means clustering method to cluster the severity of congestion in a New Delhi study. They found that their methodology can segment the roads according to the congestion severity. Similar to Pattanaik et al. work [37], Hongsakham et al. [38] applied the *k*-means clustering method to cluster the congestion levels on a particular road section in Bangkok, Thailand. Motivated by the studies of Pattanaik et al. [37] and Hongsakham et al. [38], in this work, the *k*-means clustering method was used to cluster the congestion severity based on the traffic speed data collected from smartphones in Jakarta, Indonesia.

**Table 1:** Recent studies on the effect of rainy weather on vehicle speed reduction.

Author	Year	Study Location	Road Type	Precipitation (mm/h)	Speed Drop by Weather Factor
Billot et al. [12]	2009	Paris, France	Freeway	0–2 2–3	8% 12.6%
Camacho et al. [7]	2010	Northwestern Spain	Freeway	1–2 2–5	0.8–3.0 km/h 1.4–4.6 km/h
Hou et al. [13]	2013	Irvine, United States	Highway	<2.5 2.5–7.5 >7.5	6.13% 11.38% 18.60%
Hou et al. [13]	2013	Chicago, United States	Highway	<7.5	11.90%
Hou et al. [13]	2013	Salt Lake City, United States	Highway	2.5–7.5	5.88%
Hou et al. [13]	2013	Baltimore, United States	Highway	<7.5	6.01%
Lam et al. [8]	2013	Hong Kong, China	Urban roads	0–0.5 0.5–6.5 >6.5	3.5–4.2% ∼5.7% 6.8–10.1%
Mitsakis et al. [25]	2014	Athens, Greece	Urban roads	18–27	Up to 35.4%
Hooper et al. [14]	2014	London, United Kingdom	Motorway	Wet conditions *	2.1%
Lin et al. [33]	2015	Buffalo, United States	Freeway	Wet conditions *	20%
Jägerbrand and Sjöbergh [34]	2016	Sweden	Urban roads	Wet conditions *	<1.4%
Kim and Wang [31]	2016	Brisbane, Australia	Highway	Wet conditions *	Up to 60%
Stamos et al. [26]	2016	Thessaloniki, Greece	Urban roads	0–5 5–10	0–4 km/h 4–8 km/h
Zhang et al. [15]	2018	Beijing, China	Expressway	<2.4 2.4–6.0 >6.0	3.07% 5.29% 6.64%
Kurte et al. [35]	2019	Chicago, United States	Urban roads	<5	8–20%
Ji and Shao [16]	2019	Shenzhen, China	Expressway	0-10 >10	19% >19%
Choo et al. [17]	2020	Seoul, South Korea	Urban roads	1–1.5 >1.5	<50% >50%

* No precipitation information provided.

In the traffic study, the RF method has been widely utilized in finding the contributing factors of a traffic accident. Essentially, the RF method combines many decision tree predictors where each tree carries out a random subset of features (feature bagging technique) to reduce the model variance without increasing the model bias at the same time [39]. With this superiority, RF is a popular method to rank the importance of predictive model variables. For instance, Wang et al. [40] identified that the weather and ramp geometry were the significant factors in crashes that happened on the expressway ramp in Florida, USA. Similar to Wang et al.’s work, Lee et al. [41] used the RF to investigate critical variables of traffic crashes. They found that the speed limit, collisions, and pavement condition were the significant factors influencing Florida’s traffic crash. Motivated by the studies in the literature, this study utilized RF methods to investigate the important factors regarding the WSR.

## 3. Methodology

This research proposed the STC matrix to measure and visualize the severity of the traffic speed drop caused by weather change across different periods. Then, the WSR analyses can be carried out under the STC matrix. Section 3.1 explains the proposed STC calculation, and Section 3.2 presents the proposed framework for analyzing WSR.

### 3.1. STC Matrix

**Figure 2:**
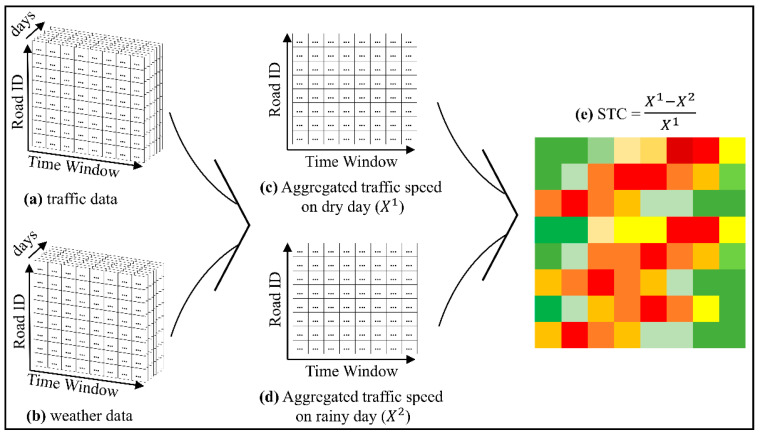
The calculation process of the proposed spatial-temporal congestion (STC) matrix.

Figure 2 illustrates the process of constructing the STC matrix. Essentially, two datasets are required: the traffic speed dataset as in Figure 2a and the weather dataset as in Figure 2b. Both datasets are summarized daily into an N×T matrix, where N is the road ID number and T is the number of time-window slots. The traffic speed and weather matrices contain the average traffic speed and precipitation at the corresponding day and time-window slots, respectively.

Figure 2c,d illustrates how to aggregate the traffic speed on dry and rainy days on the same time window based on the summarized traffic speed and weather datasets. Here, without losing the generality, the number of the aggregated matrices can be determined based on the variety of the collected weather data. Let $X^{i}\;$ be the aggregated traffic speed matrix with dimension N×T, based on the weather state *i*. If more weather states, such as dry, rainy, snowing, etc. are defined, a more aggregated traffic speed matrix $X^{i}$ can be obtained. In this work, in order to study the impact of dry and rainy seasons on traffic speed, the weather condition with two states, dry and rainy, were used (i={1, 2}).

$v_{ndt}^{i}$ is defined as the average traffic speed of road n={1,2,…,N}, at time-window t={1, 2, …, T} and day d={1, 2, …, D}, and on weather state i. Then, the average traffic speed across *D* days, can be defined as $x_{nt}^{i}$ shown in Equation (1) to be the elements of matrix $X^{i}$.

(1) $$x_{nt}^{i}=\frac{1}{D}\overset{D}{\underset{d=1}{\sum }}v_{ndt}^{i}$$

Finally, the STC matrix, illustrated in Figure 2e, can be calculated to present the speed drop scale caused by weather change. Based on Equation (2), STC is calculated by the scaled difference between $X^{1}$ and $X^{2}$ where $X^{1}$ and $X^{2}$ are traffic speed matrices under dry and rainy weather, respectively. Obviously, STC ranges from 0 to 1 because $X^{1}>X^{2}$, and both are positve values. Here, 0 indicates 0% traffic speed drop caused by the weather change. It also means the traffic speed is invulnerable to the weather. On the contrary, 1 means the road traffic completely stuck due to the raining condition.

(2) $$STC=\frac{X^{1}-X^{2}}{X^{1}}$$

**Figure 3:**
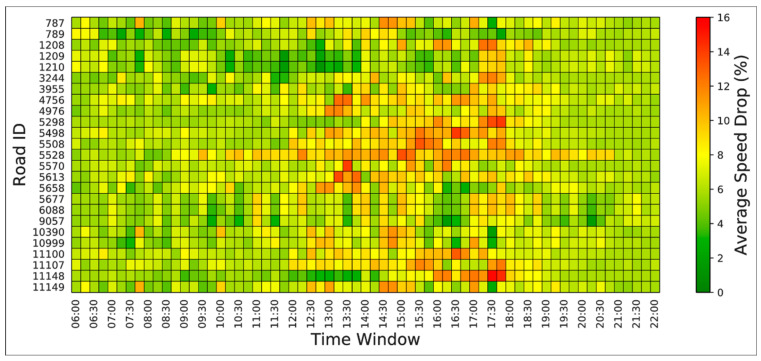
The STC matrix of the 25 representative roads in Jakarta.

Figure 3 shows an example of the STC matrix to illustrate the temporal traffic speed drop caused by weather change on Jakarta’s 25 representative roads. Basically, the x-axis and y-axis represent the time-window and road ID, respectively. The heat map colors changing from green, yellow, to red are used to indicate the relative minimum, average, and maximum vehicle speed drop caused by weather difference (dry vs. raining) under different timing. As can be seen in Figure 3, the “green” regions indicate that the weather changes from dry to rainy cause relatively minimal traffic speed drop during the nonworking hours (before 10 a.m. and after 6 p.m.). During the working hours (between 10 a.m. and 6 p.m.), the “yellow-red” regions show the severe traffic speed drop caused by rain. Through the proposed STC matrix, the roads which have a longer time with the extended speed drop can be detected. For example, road #5528 has a longer traffic speed drop period than the others (10 a.m. to 8 p.m.). Road #1209 and #1210 almost experienced no or minimal speed drop across all time windows. Based on the STC matrix, the impact of weather can be easily analyzed and used for the prediction model.

### 3.2. The Proposed Framework for Analyzing WSR Based on Sequential Clustering and Classification Analysis

This section explains the proposed framework for analyzing WSR based on sequential clustering and classification analysis. Sequential clustering and classification analysis are often used for data exploration or data analytics tasks, mainly when the classification task labels are unavailable [21]. In such a case, the clustering method is performed to generate the labels for the classification task. Then, the labels obtained from the clustering method might be the “clues” of labeling the classification task’s data afterward. Here, the problem of analyzing WSR is mainly the same as the mentioned data analytics task.

First, the clustering method can be performed to cluster the roads based on their speed drop severities. In this research, STC matrix was used as the dataset to perform K-means clustering method. By using the K-means, the roads with similar speed drop of a similar time will be clustered together. We can then further investigate the associating factors of the traffic speed drop patterns on a particular WSR cluster. Since STC matrix only considers weather change, another set of data such as road altitude above sea level, the distance to an important facility nearby, etc. can be considered for the analysis. The feature selection and data classification method can be applied to the road feature dataset combined with the cluster labels.

**Figure 4:**
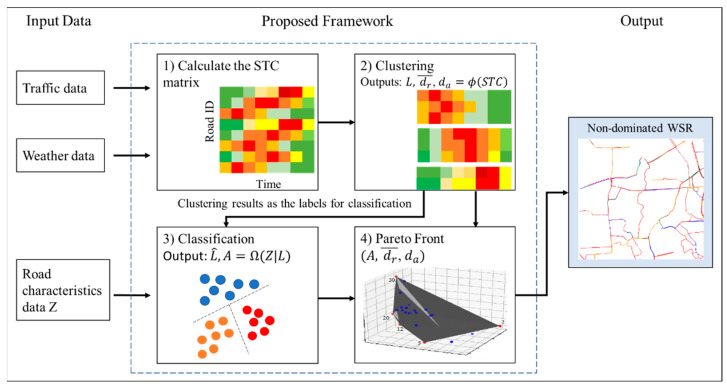
The proposed framework for weather-sensitive roads (WSR) analysis.

Figure 4 illustrates the proposed framework of identifying WSR by utilizing the STC mentioned above and the roads’ characteristics dataset [42]. As mentioned earlier, the traffic and weather datasets can be used to generate STC data. After performing K-means on STC, the generated cluster labels are appended with the road characteristic dataset. Then, the classification model is built to classify the cluster label by the road characteristics. The detailed information of clustering and classification are shown in the following sections.

- *Clustering*

After obtaining the STC matrix, the data clustering was conducted based on the STC matrix. The clustering method can be defined as a function $L,\;\bar{d_{r}},\;d_{a}=\phi \left(STC\right)$, where the clustering function ϕ processes the STC matrix as the input and resulting in three outputs: (1) labels for WSR (L), (2) the sum of squared distances between all cluster centers ($\bar{d_{r}})$, and (3) the sum of squared distances of roads to their cluster center ($d_{a}$). Given a set of roads ($X=\left\{x_{1},x_{2},\ldots ,x_{N}\right\}$) where each road is a T-dimensional real vector $x_{i}=\left\{x_{i1},x_{i2},\ldots ,x_{iT}\right\}$, *k*-means clustering aims to group elements in set X into *K* sets ($S=\left\{S_{1},\ldots ,S_{K}\right\}$), where K≤N. The *K*-means clustering’s procedure is as the following.

(1) Sample *k* roads without replacement from set *X* randomly.
(2) Assign the roads to set $\left\{S_{1},\ldots ,S_{K}\right\}$, and set the initial clusters’ centroid equal to the assigned roads.
(3) Draw a road without replacement from set X randomly ($x_{n}$).
(4) Find the nearest set to $x_{n}$, $c^{n}$, where $c^{n}$ is equal to 1 if $\begin{array}{c} \mathrm{argmin}\\ k \end{array}{\left||x_{n}-\mu_{k}|\right|}^{2}$, else 0. $c^{n}$ also represents the assignment of road $x_{n}$ to sets $S_{k},\;\forall k\in \left\{1,\ldots ,K\right\}$.
(5) Update the cluster centroids, $\mu_{k}\;:=\frac{\sum_{i=1}^{N}\left\{c^{n}=k\right\}x_{n}}{\sum_{i=1}^{N}\left\{c^{n}=k\right\}}$.
(6) Repeat steps 3–5 until convergence. Generally, the K-means clustering algorithm aims to minimize $\sum_{k=1}^{K}\sum_{i=1}^{N}{\left||\left\{c^{n}=k\right\}x_{n}-\mu_{k}|\right|}^{2}$.

The *K*-means method can group the roads based on the predetermined number for clusters *K*. It is worth mentioning that the selection of *K* can be determined by the multiobjective optimization, which will be addressed later.

- *Classification*

The classification method can be defined as a function $\hat{L}=\Omega (Z|L)$, where $\hat{L}$ are the predicted labels obtained from the classification function Ω based on the input data Z given the pregenerated L from the clustering method. Following the previous work, this study’s performance metric is the Hamming loss, as this loss function is standard for the multilabel classification problem [43]. Hamming loss converts the class labels into unique binary strings and calculates the loss generated based on exclusive disjunction (XOR) operation between the actual and predicted class labels’ binary strings. The Hamming loss follows Equation (3), where A, N, M, ⊗, $y_{ij}$, ${\hat{y}}_{ij}$ are the model accuracy, number of instances, length of the binary strings, XOR operation, actual and predicted class label *j* in data instance i, respectively.

(3) $$A=1-\frac{1}{\left|N\right|\cdot \left|M\right|}\overset{L}{\underset{i=1}{\sum }}\overset{M}{\underset{j=1}{\sum }}⊗\left(y_{ij}-{\hat{y}}_{ij}\right)$$

The proposed framework considers the RF method for the classification task [39]. The RF model is an ensemble model of B classification trees (CT). Essentially, RF method generates the prediction based on majority prediction results of the CT. The training of RF algorithm for the WSR study is shown as the following.

(1) Sample B observations from Z with replacement.
(2) Sample randomly m of the p independent variables.
(3) Find the split $s_{Q}$ among all possible split’s location for the Q-th variable, Q=1,2,…,m, to classify the given road label and identifies the cut point *p*′.
(4) Split the data at the node $s_{Q}$, assign the data to the left descendant of the decision tree if $x_{bQ}\ldots p^{\prime }$, and to the right descendant if $x_{bQ}>p$′, where b=1,2,≤,B.
(5) Repeat steps 2–4 until the tree grows maximally.

- *Multiobjective Optimization*

As mentioned earlier, how to determine the number of clusters for *K*-means is a challenging question. Smaller K might not represent the variety of the WSR, and larger K (lager number of labels) might escalate the problem complexity, especially for the classification task. In terms of objective functions, different *K* will generate different results of the clustering problem ($\bar{d_{r}}$ and $d_{a}$) and the classification problem (A). Therefore, the multiobjective optimization method is used in the proposed framework to find the nondominated *K* to maximize $\bar{d_{r}}$, reciprocal of $d_{a}$, and A.

Here, we applied the Pareto front method [24] for solving the above-mentioned multiobjective optimization. Given a set of possible solutions $S^{d}$, which is the number of classes for clustering (see the dots in the Pareto plot in Figure 4), and d is the number of considered objectives. Given two vectors in the objective space, $s^{1}\in S^{d}$ and $s^{2}\in S^{d}$. The vector $s^{1}$ is said to dominate $s^{2}$ if and only if $s_{i}^{1}\geq s_{i}^{2},\;\forall i\in \left\{1,\ldots ,d\right\}$, ∃j∈{1,…,d}. In other words, the solutions in vector $s^{1}$ do not dominate each other in $s^{1}$, and they dominate the solutions in vector $s^{2}$. The solutions in vector $s^{1}$ are known as nondominated solutions. Based on the process of finding the nondominated solutions $s^{1}$, the multiple *K* can be selected from vector $s^{1}$. The optimal *K* can be further chosen based on the comparison among the criteria. The experiment of the real-world data analysis shown in Section 4 will provide a detailed example of choosing *K* from the nondominated solutions.

## 4. Results

### 4.1. Dataset

This paper considers three datasets for studying WSR: Dataset #1 is a traffic speed dataset based on smartphone sensors data, Dataset #2 is a weather dataset, and Dataset #3 contains roads’ characteristics. In this research, Dataset #1, provided by the Jakarta Smart City Department, a research unit under the Government of Jakarta, Indonesia, contains the traffic speed data in Jakarta, collected from November 2017 to October 2018. The traffic speed data contains the GPS information, which includes the real-time information coordinate and vehicle speed information from citizens’ smartphone sensors in Jakarta while they are traveling using cars and motorcycles. The size of Dataset #1 is 600 Gigabytes with more than two billion traffic speed records. Table 2 shows the example of the processed Dataset #1, and more detailed information of the preprocessing data can be found in the previous work [3]. Four main attributes in Dataset #1 were used to build Jakarta’s traffic speed: the time information, location latitude, location longitude, and the recorded speeds. The traffic speed used in this case is motor vehicle speed (motorcycles and cars) only. We reconstruct the attributes into geographic information systems (GIS) data representation by matching the attributes with the GIS information of roads in Jakarta in OpenStreetMap [42].

Dataset #2, collected from WorldWeatherOnline^®^ [44], is the weather information of Jakarta City between November 2017 and October 2018. Since the effect of rain against the traffic speed is the main research topic, we consider only two weather states: dry and rainy. Dataset #2 contains the rain intensity and the associated date and time information. The examples of Dataset #2 can be seen in Table 3.

Dataset #3, obtained from OpenStreetMap [42], contains the roads’ characteristic information in Jakarta, as shown in Table 4. The obtained information regarding a particular road and its surroundings includes the length, width, number of lanes, types, the altitude of the streets, and distances to the nearest public areas, such as schools, mosques, and shopping malls. The distance between a particular road segment and the site (shopping malls, mosques, schools) is measured based on the Euclidean distance of the corresponding coordinates. We later used these features in Table 4 as the RF method’s predictor variables to predict the speed drop.

**Figure 5:**
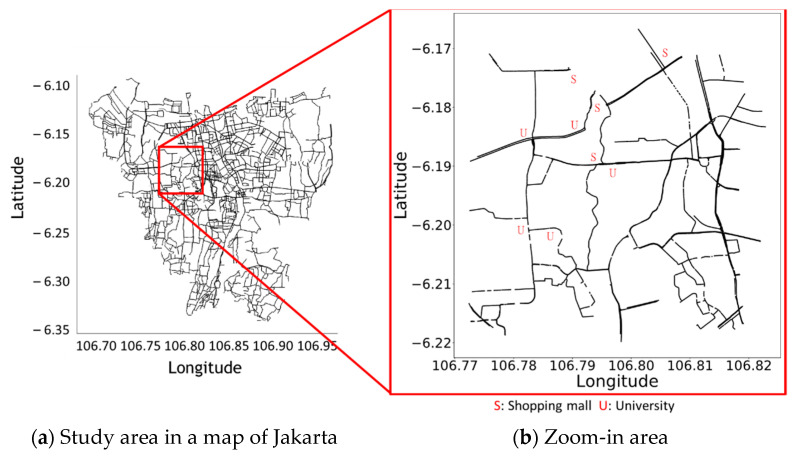
The case study area in west Jakarta.

In this paper, the study region, a 5 × 5 km-square area of nonresidential roads in west Jakarta, as marked in red-box in Figure 5a, is used to represented a case study. Figure 5b is the zoom-in of the red-box on the left-hand side. The reason of using this area is because the selected location can represent the condition in Jakarta’s roads in general. In the chosen 5 × 5 km-square area, there are 906 roads, three large shopping mall complexes, business districts, three local universities, the entrance and exit gates of a highway toll road, a commuter station, and plenty of wide and narrow streets. In fact, this 5 × 5 km-square area is the core area of Jakarta city, representing a common urban area in a metropolis of a developing country without losing the generality. The traffic data were aggregated into fifteen-minute intervals from 6 a.m. to 10 p.m.

Please note that this research particularly emphasizes urban traffic on weekdays; therefore, we omitted the traffic data on holidays and weekends. To anticipate the error in the GPS reading, based on the research work suggestion in [45], we included all of the traffic data within 10 m in calculating the average traffic speed. In this research, only the traffic speed lower than 10 km/h was used for investigation because the speed greater than 10 km/h was considered the normal traffic based on the Jakarta government’s definition. We assumed all factors except in Table 4 that can create traffic congestion, such as traffic incident, as the implicit factors in the WSR analysis.

We tested the significance of the average traffic speed based on the paired t-test between the dry and rainy days. Our preliminary results show that in 58,890 combinations (906 roads times 65 window slots), 90% of combinations are statistically significant with a significance level of 0.05. It also means that most of the STC matrix data showing the speed on a dry day is higher than it on a rainy day has been statistically verified. The remaining 10% are nonsignificant results are mainly due to the lack of traffic data during the night time and on the less traveled road.

### 4.2. Selection of K for Clustering

This section reports the process of selecting K based on Pareto front optimization of $\bar{d_{r}}$, reciprocal of $d_{a}$ and A, where the three objectives were obtained from the following experiments. As explained earlier in Section 3, the Pareto front method determines the best *K* with considering the optimization of the objectives of both clustering and classification methods: $\bar{d_{r}}$, reciprocal of $d_{a}$ and A. This paper assumes that finding the best-fit *K* from the nondominated solutions based on objective functions can lead to a better WSR result.

**Figure 6:**
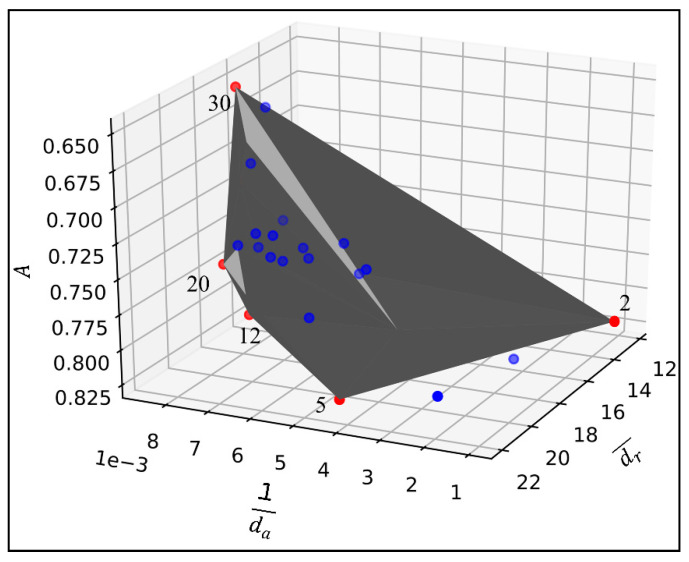
A three-dimension visualization of the Pareto surface associated with different *K*. The red dots indicate the nondominated solutions, and the blue dots indicate the dominated solutions. The grey surface is the Pareto frontier surface.

We simulated K-means clustering algorithm with *K* from 2 to 30 and recorded the results of $\bar{d_{r}}$ and $d_{a}$. For each *K*, the prediction accuracies of the RF method A were stored together with the $\bar{d_{r}}$ and $d_{a}$. Then, the selection of best *K* was conducted based on the Pareto Front method. Figure 6 visualizes the solutions under Pareto surface of the reciprocal of $d_{a}$ (x-axis), $\bar{d_{r}}$ (y-axis), and A (z-axis). The grey surface in Figure 6 represents the Pareto frontier area. The edges of the Pareto frontier are the nondominated solutions (red dots) which are not dominating each other in the same solution set. The blue dots are the solutions that are dominated by red dots. From Figure 6, the five nondominated solutions are K= 2, 5, 12, 20, and 30.

Table 5 lists the *K*, and the associated $\bar{d_{r}}$ reciprocal of $d_{a}$, and A of the nondominated solutions as in Figure 6. Among the five nondominated solutions, the *K* with relatively higher A is preferred because lower A means the classifier has poor performance in predicting the WSR label using the prediction variables in Table 4. It also means the lower prediction accuracy *A* implies that the road characteristics cannot help on predicting the speed drop level of WSR. Therefore, K=2 and K=5 are the preferred candidates with relatively higher level of A, 0.822 and 0.817, respectively.

When comparing the solutions with K=2 and K=5, the $\bar{d_{r}}$ and the reciprocal of $d_{a}$ of solution K=5 are 22.000 and 0.004, respectively, which are higher than it in solution k=2 (higher is better). Based on this comparison, this study considers the solution K=5 as the best fit solution for representing WSR.

**Figure 7:**
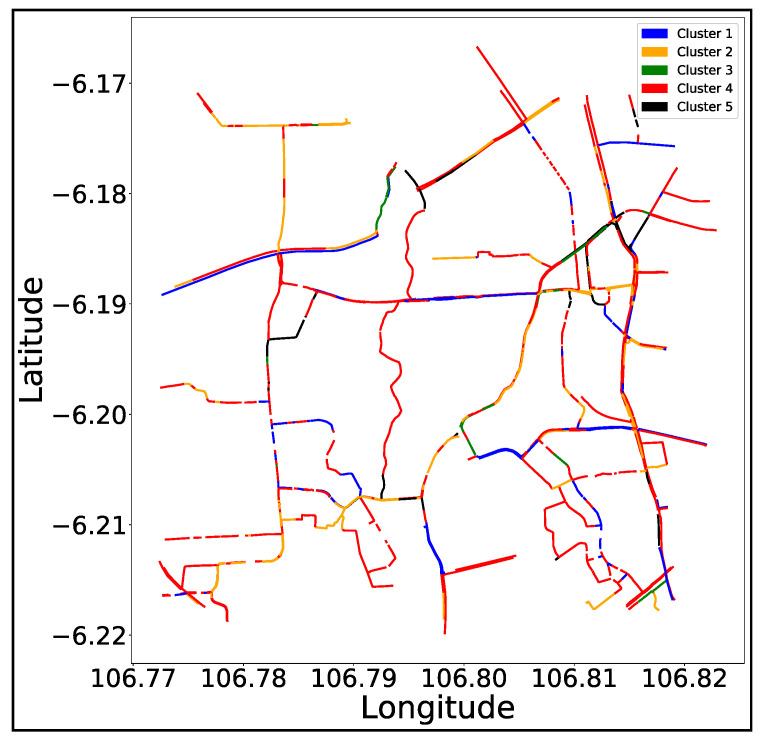
The road representation of the clustering results of the experimented area.

Figure 7 shows the clustering results using K=5. In Figure 7, the blue, yellow, green, red, and black colors indicate the roads in clusters #1, #2, #3, #4, and #5, respectively. Also, the number of roads of each cluster was 92, 159, 573, 61, and 21 for cluster #1, #2, #3, #4, and #5, respectively. Generally, #3 and #5 have relatively fewer roads than clusters #1, #2, and #4, which acquire most of the roads in the experimented area. 

### 4.3. The WSR Analysis

- Speed Drop Pattern

**Figure 8:**
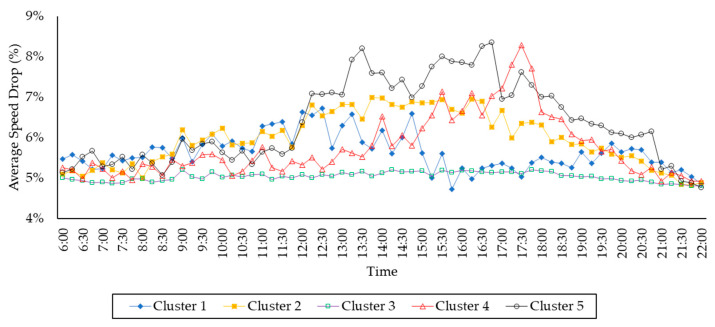
The average speed drop of five clusters.

Figure 8 shows the average speed drop in kilometers per hour (y-axis) of five clusters of WSR over time (x-axis). Please note that the traffic speed drop is due to weather change (dry vs rainy). Figure 8 uses the same color indicators shown in Figure 7 to represent the road clusters, with additional shape indicators following by the blue diamond, orange X, green rectangle, red triangle, and black circle.

In general, Jakarta roads experience at least a 4.7% speed drop when the weather changes from dry to rainy. Obviously, each road cluster has different speed drop patterns over time. For example, the average speed drop in cluster #3 is approximately closer to 5%. Except in cluster #3, we can observe the speed drop patterns of all clusters in four time-frames: (1) from 6 a.m. to 12 p.m. (morning), (2) from 12 p.m. to 4 p.m. (early afternoon), (3) from 4 p.m. to 8 p.m. (late afternoon), and (4) from 8 p.m. to 10 p.m. (night). In the morning, the speed drop of cluster #2 steadily increases from 5% to 6.5% over time. The speed drop of cluster #2 is stable at around 6.5% in the early afternoon and declines until 4.9% during the late afternoon and night time.

The speed drop pattern of cluster #1 is relatively unique compared to Clusters #2 and #3. The speed drop of cluster #1 increases from 5.2% to 6.6% in the morning time and declines to 5% in the early afternoon time. Interestingly, the speed drop increases for the second time in the later afternoon to 5.8%, before it declines for the second time during the night time to 4.8%.

In cluster #4, the speed drop is relatively constant at around 5.1% in the morning time. The speed drop begins to increase to 7.1% in the early afternoon time. During the later afternoon, the speed drop increases quickly to 8.3% at 5.30 p.m. and declines after that to just 5.7% at 8 p.m. The speed drop keeps decreasing to 4.8% during the night.

Among all clusters, in general, cluster #5 has a higher traffic speed drop. The speed drop of cluster #5 increases slowly from 5.2% to 5.7% during the morning time. During the early afternoon, the speed drop jumps to 8.2% at 1.30 p.m. and stable at around 7.5%. The speed drop increases to 8.35% at 4.45 p.m. and steadily decreases to 6.1% during the late afternoon. The speed drop continues to decline to 4.75% during the night time. Between 12 to 6 p.m., the speed drop of cluster #5 is between 7% and 8.35%, and this is the highest speed drop compared to other clusters, where the speed drops are mostly below 7%, except the anomaly in cluster #4 between 5.30 p.m. and 6.30 p.m.

The further investigation on how the characteristics of these road clusters are associated with the corresponding speed drop patterns caused by raining is presented in the following subsection.

- Road Characteristics Associated with WSR

**Figure 9:**
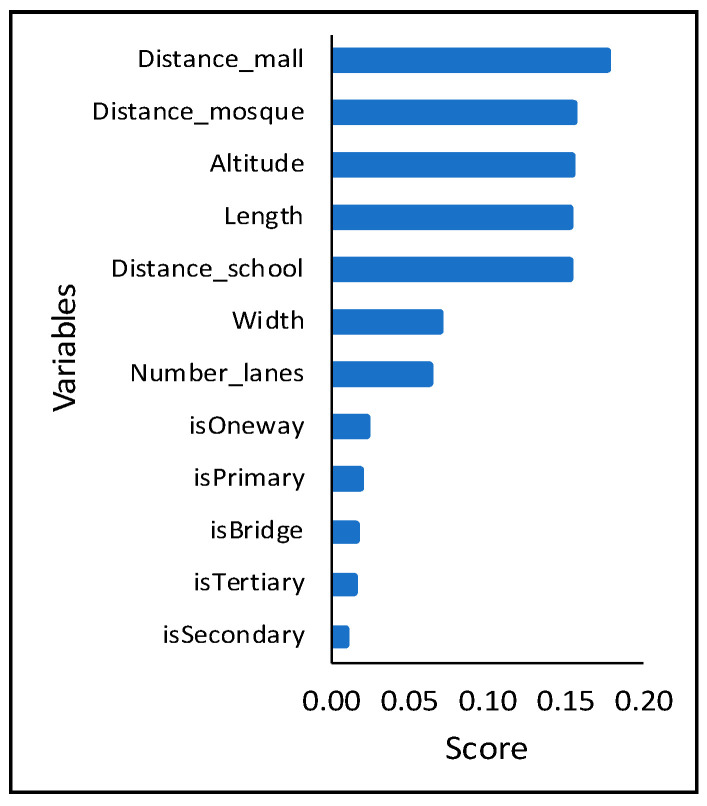
The characteristics of WSR.

Figure 9 lists the road characteristics with their significance, obtained from RF classifier, from top to bottom based on Table 4. As shown in Figure 9, the most crucial factor, shown on the top, is the distance to the nearest shopping mall, with an importance score of 0.175 from RF method. Other essential variables are the distance to the nearest mosque and school, the altitude, and the roads’ length with the importance score of 0.154, 0.151, 0.153, and 0.152. The roads’ width and the number of lanes are also crucial, with the importance score of 0.081 and 0.072, respectively.

It is interesting to associate the variables in Figure 9 with the road clusters to describe the WSR. Table 6 shows the significant road characteristics in Figure 9 against the five WSR clusters. Based on Table 6, cluster #3 has the highest average altitude compared to other clusters. It can be the reason why the impact of weather changes on cluster #3 is considerably stable, around 5%, from morning until night. Cluster #3 also has the smallest average road length and width, which means the roads are relatively shorter and narrow than other clusters. Intuitively, the number of vehicles in cluster #3 might be less than other clusters; thus, the impact of weather change is relatively constant.

Cluster #1 consists of relatively long roads, mostly single and two-lane roads, and relatively far from schools. Unlike cluster #1, cluster #2 has shorter and broader roads with higher altitudes. Cluster #2 roads are also relatively far from public areas, and have speed drops peaks in the afternoon.

Cluster #4 has relatively low altitude and is very close to mosques and schools that are essential facilities in Indonesia’s citizen life. The highest average speed drop on these roads occurs during the late afternoon (5.30 p.m. to 6.30 p.m.). Based on the Maghrib prayer schedule, the period of 5.30 p.m. to 6.30 p.m. is the typical prayer time in a day because it is the only between sunset and the beginning of the night. As a result, the speed drop on this cluster accumulating to be the highest between the prayer time.

The roads in cluster #5 are closer to shopping malls and relatively far from mosques and schools than other clusters. Uniquely in Jakarta, shopping malls are the main tourist sites and the primary destination for family recreation. Furthermore, people in Jakarta also hang out at shopping malls [44]. The shopping malls in Jakarta usually open at 11 a.m. and close at 10 or 11 p.m. Therefore, these roads experience the highest average speed drop during the shopping malls’ operational time (see Figure 8).

In short, based on the proposed data analysis framework, the speed drop between the day and rainy weather conditions was used to present the traffic condition of roads in Jakarta. Then, the sequential clustering and classification process was conducted to cluster the roads and search for associating the characteristics of roads. The case study in real-world data in Jakarta shows the framework is able to not only identify WSR with significant factors regarding the speed drop but also provide insights useful for city traffic management.

## 5. Conclusions

WSR identification and analysis are crucial for city development. Setting the higher priority of maintenance on WSR over the non-WSR can be more cost-effective in reducing traffic congestion. Also, providing locations of WSR to road users can help drivers bypass the WSR when it is about to rain. Especially for developing countries, such as southern Asian countries where the mass transportation system is limited or under construction, millions of motorcyclists can count on the information of WSR to enhance the mobility of transportation on rainy days.

In this research, to fill the research gap, Jakarta’s traffic pattern was studied as a representative example of the metropolis in the developing country. Because of the inadequate sewer systems in Jakarta, rains often create a lot of sudden traffic on the roads that are traffic-free in dry weather. This study focuses on identifying and analyzing the causes of WSR using machine learning methods based on smartphone sensors, weather, and road characteristics datasets. A framework consisting of sequential clustering and classification tasks was proposed. We first introduced the STC matrix to representing the roads’ average speed drop caused by the weather changed from dry to rainy. Then, the STC matrix was clustered by using *K*-means clustering method. The clustering labels were used as the prediction labels for the classification tasks. In this research, the RF method was used in the classification tasks to investigate the associating causes of WSR based on the given dataset. The Pareto front method was used to select the *K*, based on the objectives of both the clustering and classification methods.

The experimental results show that *K = 5* is chosen to represent the WSR in Jakarta. Based on this study, the unique speed drop patterns of road clusters can be observed. For example, roads in cluster #4 face a significant speed drop during the late afternoon, while the opposite effect showed in cluster #1. Using the RF method, seven leading factors of WSR in Jakarta were found out: the distances to (1) shopping malls, (2) mosques, (3) schools, and the roads’ (4) altitude, (5) length, and (6) width, and (7) the number of lanes, with the importance scores of 0.175, 0.154, 0.151, 0.153, 0.152, 0.081 and 0.072, respectively.

The main contribution of this work is to propose the framework which can be used to assess the impact of weather change against the road traffic speed. Without losing the generality, the proposed analysis framework can be practically applied in many other weather changes, such as fog and snow. Since the current dataset does not contain precipitation information, in the future, investigating how precipitation affects the speed drop of WSR could be the next task. Moreover, incorporating more parameters in the WSR study, such as traffic incidents, is worth future study. Last but not least, other clustering and classification methods, such as the fuzzy C-means clustering algorithm, can be integrated to extend the proposed framework for finer clusters.

## Figures and Tables

**Table 2:** Examples of Dataset #1.

Longitude	Latitude	Speed (km/h)	Level	Time
106.782318	−6.198835	2.91	3	17 November 2017 00:18:56
106.899734	−6.218775	1.32	4	16 November 2017 23:34:48
106.782875	−6.333290	3.44	3	17 November 2017 00:04:58
106.825172	−6.187048	2.31	3	17 November 2017 00:02:35
106.738625	−6.126846	3.51	3	17 November 2017 00:11:52

**Table 3:** Examples of Dataset #2.

Date	Month	Time	Weather	Temperature	Precipitation	Year
Tue 01	Aug	0:00	Patchy rain possible	29	0	2017
Tue 01	Aug	3:00	Partly cloudy	30	0	2017
Tue 01	Aug	6:00	Cloudy	33	0	2017
Tue 01	Aug	12:00	Sunny	38	0	2017
Tue 01	Aug	18:00	Clear	35	0	2017

**Table 4:** Characteristics of the road segments.

Name	Description
isBridge	1—If the road is a bridge, 0—otherwise
isOneway	1—If the road applies the one-way policy, 0—otherwise
isPrimary	1—If the road is the main road, 0—otherwise
isSecondary	1—If the road is the secondary road, 0—otherwise
isTertiary	1—If the road is the tertiary road, 0—otherwise
Length	Length of the road in meter unit
Width	Width of the road in meter unit
Number_lanes	Number of lanes
Type_road	Three types: primary, secondary, tertiary
Altitude	The road’s altitude (meter) above the sea
Distance_school	Distance (meter) to the nearest school to the road segment
Distance_mosque	Distance (meter) to the nearest mosque to the road segment
Distance_mall	Distance (meter) to the nearest mall to the road segment

**Table 5:** The nondominated solutions on the Pareto surface.

k	$\bar{d_{r}}$	$\mathrm{Reciprocal}\;\mathrm{of}\;d_{a}$	A
2	12.267	0.001	0.822
5	22.000	0.004	0.817
12	20.831	0.006	0.770
20	19.454	0.007	0.740
30	16.863	0.008	0.652

**Table 6:** Characteristics of WSR clusters.

Variables	Cluster 1	Cluster 2	Cluster 3	Cluster 4	Cluster 5
Distance_mall	975.1	1256.5	1069.8	966.5	650.8
Distance_mosque	149.3	178.7	146.2	127.9	192.7
Length	150.2	98.7	91.1	151.7	135.2
Width	6.26	6.68	6.23	7.00	8.33
Number_lanes	1.87	2.57	2.01	2.10	2.48
Altitude	10.29	10.32	10.69	8.68	10.02
Distance_school	232.3	203.3	192.3	174.1	240.4

## Data Availability

The data used in this research is owned by Jakarta Smart City. Any request regarding the data should contact Jakarta Smart City.

## References

[1] Truitt A. (2008). On the back of a motorbike: Middle-class mobility in Ho Chi Minh City, Vietnam. Am. Ethnol..

[2] Joewono T.B., Tarigan A.K.M., Susilo Y.O. (2016). Road-based public transportation in urban areas of Indonesia: What policies do users expect to improve the service quality?. Transp. Policy.

[3] Tampubolon H., Yang C.-L., Chan A.S., Sutrisno H., Hua K.-L. (2019). Optimized CapsNet for Traffic Jam Speed Prediction Using Mobile Sensor Data under Urban Swarming Transportation. Sensors.

[4] Desk N. (2017). Jakarta foots US$5b annual bill for traffic jams: Minister. The Jakarta Post.

[5] Haider A.A. Traffic Jam: The Ugly Side of Dhaka’s Development. Last Modified on 13 May 2018. https://www.thedailystar.net/opinion/society/traffic-jam-the-ugly-side-dhakas-development-1575355.

[6] Japan International Cooperation Agency (2018). JICA to Help Philippines Ease Traffic Congestion in Metro Manila.

[7] Camacho F.J., García A., Belda E. (2010). Analysis of Impact of Adverse Weather on Freeway Free-Flow Speed in Spain. Transp. Res. Rec. J. Transp. Res. Board.

[8] Lam W.H.K., Tam M.L., Cao X., Li X. (2013). Modeling the Effects of Rainfall Intensity on Traffic Speed, Flow, and Density Relationships for Urban Roads. J. Transp. Eng..

[9] Sathiaraj D., Punkasem T.-O., Wang F., Seedah D.P.K. (2018). Data-driven analysis on the effects of extreme weather elements on traffic volume in Atlanta, GA, USA. Comput. Environ. Urban Syst..

[10] The Department of Transportation, Taipei City Government Taipei City ATIS Web. https://its.taipei.gov.tw/?lang=en.

[11] Marino F., Leccese F., Pizzuti S. (2017). Adaptive Street Lighting Predictive Control. Energy Procedia.

[12] Billot R., Faouzi N.-E.E., Vuyst F.D. (2009). Multilevel Assessment of the Impact of Rain on Drivers’ Behavior: Standardized Methodology and Empirical Analysis. Transp. Res. Rec. J. Transp. Res. Board.

[13] Hou T., Mahmassani H.S., Alfelor R.M., Kim J., Saberi M. (2013). Calibration of Traffic Flow Models Under Adverse Weather and Application in Mesoscopic Network Simulation. Transp. Res. Rec. J. Transp. Res. Board.

[14] Hooper E., Chapman L., Quinn A. (2014). The impact of precipitation on speed–flow relationships along a UK motorway corridor. Theor. Appl. Climatol..

[15] Zhang W., Li R., Shang P., Liu H. (2019). Impact Analysis of Rainfall on Traffic Flow Characteristics in Beijing. Int. J. Intell. Transp. Syst. Res..

[16] Ji X., Shao C. (2019). Modeling and Analyzing Free-Flow Speed of Environment Traffic Flow in Rainstorm Based on Geomagnetic Detector Data. Ekoloji.

[17] Choo K.-S., Kang D.-H., Kim B.-S. (2020). Impact Assessment of Urban Flood on Traffic Disruption using Rainfall–Depth–Vehicle Speed Relationship. Water.

[18] Akbar A. Berapa Kepadatan Penduduk DKI Jakarta saat ini?. https://statistik.jakarta.go.id/berapa-kepadatan-penduduk-dki-jakarta-saat-ini/.

[19] TomTom Traffic Index 2019. https://www.tomtom.com/en_gb/traffic-index/ranking/.

[20] Nunez K. Does Your Jogging Speed Feel Right?. https://www.healthline.com/health/average-jogging-speed.

[21] Yang C.-L., Quyen N.T.P. (2018). Data analysis framework of sequential clustering and classification using non-dominated sorting genetic algorithm. Appl. Soft Comput..

[22] Hartigan J.A., Wong M.A. (1979). Algorithm AS 136: A K-Means Clustering Algorithm. J. R. Stat. Society. Ser. C (Appl. Stat.).

[23] Ho T.K. Random Decision Forests. Proceedings of the 3rd International Conference on Document Analysis and Recognition.

[24] Veldhuizen D.A.V., Lamont G.B. Evolutionary computation and convergence to a pareto front. Proceedings of the Genetic Programming 1998 Conference (GP-98).

[25] Mitsakis E., Stamos I., Diakakis M., Grau J.M.S. (2014). Impacts of High-Intensity Storms on Urban Transportation: Applying Traffic Flow Control Methodologies for Quantifying the Effects. Int. J. Environ. Sci. Technol..

[26] Stamos I., Grau J.M.S., Mitsakis E., Aifadopoulou G. (2016). Modeling Effects of Precipitation on Vehicle Speed. Transp. Res. Rec. J. Transp. Res. Board.

[27] Kidando E., Kitali A.E., Lyimo S.M., Sando T., Moses R., Kwigizile V., Chimba D. (2019). Applying Probabilistic Model to Quantify Influence of Rainy Weather on Stochastic and Dynamic Transition of Traffic Conditions. J. Transp. Eng. Part A Syst..

[28] Yao Y., Wu D., Hong Y., Chen D., Liang Z., Guan Q., Liang X., Dai L. (2020). Analyzing the Effects of Rainfall on Urban Traffic-Congestion Bottlenecks. IEEE J. Sel. Top. Appl. Earth Obs. Remote Sens..

[29] Dunne S., Ghosh B. (2013). Weather Adaptive Traffic Prediction Using Neurowavelet Models. IEEE Trans. Intell. Transp. Syst..

[30] Chen Y.-Y., Lv Y., Li Z., Wang F.-Y. Long short-term memory model for traffic congestion prediction with online open data. Proceedings of the 2016 IEEE 19th International Conference on Intelligent Transportation Systems (ITSC).

[31] Kim J., Wang G. (2016). Diagnosis and Prediction of Traffic Congestion on Urban Road Networks Using Bayesian Networks. Transp. Res. Rec. J. Transp. Res. Board.

[32] Koesdwiady A., Soua R., Karray F. (2016). Improving Traffic Flow Prediction With Weather Information in Connected Cars: A Deep Learning Approach. IEEE Trans. Veh. Technol..

[33] Lin L., Ni M., He Q., Gao J., Sadek A.W. (2015). Modeling the Impacts of Inclement Weather on Freeway Traffic Speed: Exploratory Study with Social Media Data. Transp. Res. Rec. J. Transp. Res. Board.

[34] Jägerbrand A.K., Sjöbergh J. (2016). Effects of weather conditions, light conditions, and road lighting on vehicle speed. SpringerPlus.

[35] Kurte K., Ravulaparthy S., Berres A., Allen M., Sanyal J. (2019). Regional-scale Spatio-Temporal Analysis of Impacts of Weather on Traffic Speed in Chicago using Probe Data. Procedia Comput. Sci..

[36] Liu D. (2017). Design of Wireless Sensor Intelligent Traffic Control System based on K-means Algorithm. Rev. De La Fac. De Ing..

[37] Pattanaik V., Singh M., Gupta P., Singh S. Smart real-time traffic congestion estimation and clustering technique for urban vehicular roads. Proceedings of the 2016 IEEE Region 10 Conference (TENCON).

[38] Hongsakham W., Pattara-atikom W., Peachavanish R. Estimating road traffic congestion from cellular handoff information using cell-based neural networks and K-means clustering. Proceedings of the 2008 5th International Conference on Electrical Engineering/Electronics, Computer, Telecommunications and Information Technology.

[39] Breiman L. (2001). Random forests. Mach. Learn..

[40] Wang L., Shi Q., Abdel-Aty M. (2015). Predicting Crashes on Expressway Ramps with Real-Time Traffic and Weather Data. Transp. Res. Rec. J. Transp. Res. Board.

[41] Lee J., Nam B., Abdel-Aty M. (2015). Effects of Pavement Surface Conditions on Traffic Crash Severity. J. Transp. Eng..

[42] OpenStreetMap Data Working Group https://planet.osm.org.

[43] Li T., Zhang C., Zhu S. Empirical Studies on Multi-label Classification. Proceedings of the 18th IEEE International Conference on Tools with Artificial Intelligence (ICTAI’06).

[44] World Weather Online. https://www.worldweatheronline.com/developer/api/local-city-town-weather-api.aspx.

[45] Merry K., Bettinger P. (2019). Smartphone GPS accuracy study in an urban environment. PLoS ONE.

